# Figure‐of‐Eight Stabilizing Shoulder Taping Combined With Ultrasound‐Guided Exercise Therapy for Traumatic Recurrent Massive Rotator Cuff Tear: A Case Report

**DOI:** 10.1002/ccr3.71932

**Published:** 2026-01-24

**Authors:** Takaki Aruga, Masashi Kawabata, Hidenori Yao

**Affiliations:** ^1^ Department of Rehabilitation Sassa Orthopedic and Dialysis Clinic Tokyo Japan; ^2^ Department of Rehabilitation Kitasato University School of Allied Health Sciences Sagamihara Japan; ^3^ Department of Orthopedic Surgery Hokuso Shiroi Hospital Chiba Japan

**Keywords:** bandages, exercise therapy, rotator cuff injuries, shoulder joint, ultrasonography

## Abstract

Figure‐of‐eight stabilizing shoulder taping immediately reduced pain and restored joint centering in a patient with traumatic recurrent massive rotator cuff tear. When combined with real‐time ultrasound‐guided exercise therapy, it facilitated selective posterior cuff activation and enabled short‐term functional recovery, offering a feasible conservative option for patients declining surgery.

**Abbreviations**
ADL: Activities of daily living
JOA: Japanese Orthopedic Association
MRCT: Massive rotator cuff tears
MRI: Magnetic resonance imaging
NRS: Numerical rating scale
RSA: Reverse shoulder arthroplasty

## 1 Introduction

Massive rotator cuff tears (MRCT) are a major cause of functional disability and limitations in activities of daily living (ADL) among older adults. In a large ultrasound screening survey of a rural Japanese population, the prevalence of MRCT among all full‐thickness tears was high across age groups—43.8% in individuals in their 60s, 45.1% in their 70s, and 43.9% in their 80s [1].

Conservative treatment may provide benefit in some cases; however, approximately 36% of patients eventually require surgery [2], highlighting the limited effectiveness of nonoperative management. In MRCT, loss of cuff integrity compromises dynamic glenohumeral stability, making it difficult to maintain a centered humeral head and restore functional movement. Among the surgical options, reverse shoulder arthroplasty (RSA) remains the gold standard [3]. RSA improves all components of the Constant score and increases forward elevation by an average of 66° [4]. However, RSA carries notable risks—including infection, dislocation, and implant loosening [5]—and may not be feasible for older adults with multiple comorbidities or for those who decline surgery [6].

Recently, Kawabata et al. reported that a novel figure‐of‐eight stabilizing shoulder taping applied to baseball pitchers with marked anterior instability restrained anterior humeral head translation, providing immediate pain relief and improving throwing performance [7]. The authors suggested that its effects were mediated not only by mechanical stabilization of the glenohumeral joint but also by motor learning in a centered joint position.

Herein, we report the case of an older adult patient with traumatic recurrent MRCT who declined surgical treatment. Application of this taping technique, combined with real‐time ultrasound feedback to facilitate activation of the residual cuff musculature, led to immediate improvement in pain and active elevation, enabling the patient to resume recreational activity.

## 2 Case History and Examination

The patient was a woman in her 70s who sustained a left shoulder injury after a fall. She was right‐hand dominant, and the affected shoulder was the left, non‐dominant side. Magnetic resonance imaging (MRI) revealed a massive rotator cuff tear consistent with postoperative retear. Her medical history included an ipsilateral rotator cuff tear and shoulder dislocation in her 50s, for which she had undergone rotator cuff repair and distal clavicle resection. Considering her advanced age and low expectations of the surgical outcomes, she declined revision surgery and opted for conservative management. The treatment goals were to achieve independence in activities of daily living and return to recreational table tennis once a week. Physical therapy was initiated 6 weeks post‐injury.

At baseline, she reported difficulty with activities of daily living, particularly grooming and dressing. Neurological screening revealed no abnormalities. Passive shoulder elevation was preserved; however, active elevation was severely limited and accompanied by pain, meeting the clinical criteria for pseudoparalysis.

Clinical examination revealed a positive lag sign and a positive belly‐press test, indicating impaired posterior rotator cuff function and reduced subscapularis strength on the affected side.

**FIGURE 1:**
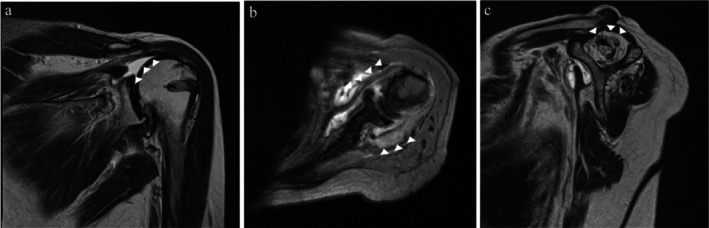
Magnetic resonance imaging findings. (a) Massive tear of the supraspinatus tendon consistent with the Cofield classification (arrowhead). (b) Attenuation and discontinuity of the infraspinatus and subscapularis tendons (arrowhead). (c) Fatty infiltration of the supraspinatus muscle consistent with Goutallier stage IV disease (arrowhead).

Plain radiography revealed superior humeral head migration, and MRI revealed a massive tear of the supraspinatus tendon consistent with Cofield's classification, attenuation and discontinuity of the infraspinatus and subscapularis tendons, and fatty infiltration of the supraspinatus, classified as Goutallier stage IV (Figure 1).

Upon the initial clinical assessment, active forward elevation was limited to 85° and abduction to 70°, with motion‐related pain rated 8 on the numerical rating scale (NRS). The Japanese Orthopedic Association (JOA) shoulder score was 38 points, which is markedly lower than the values typically reported in patients with satisfactory postoperative outcomes (> 80 points) [8].

**FIGURE 2:**
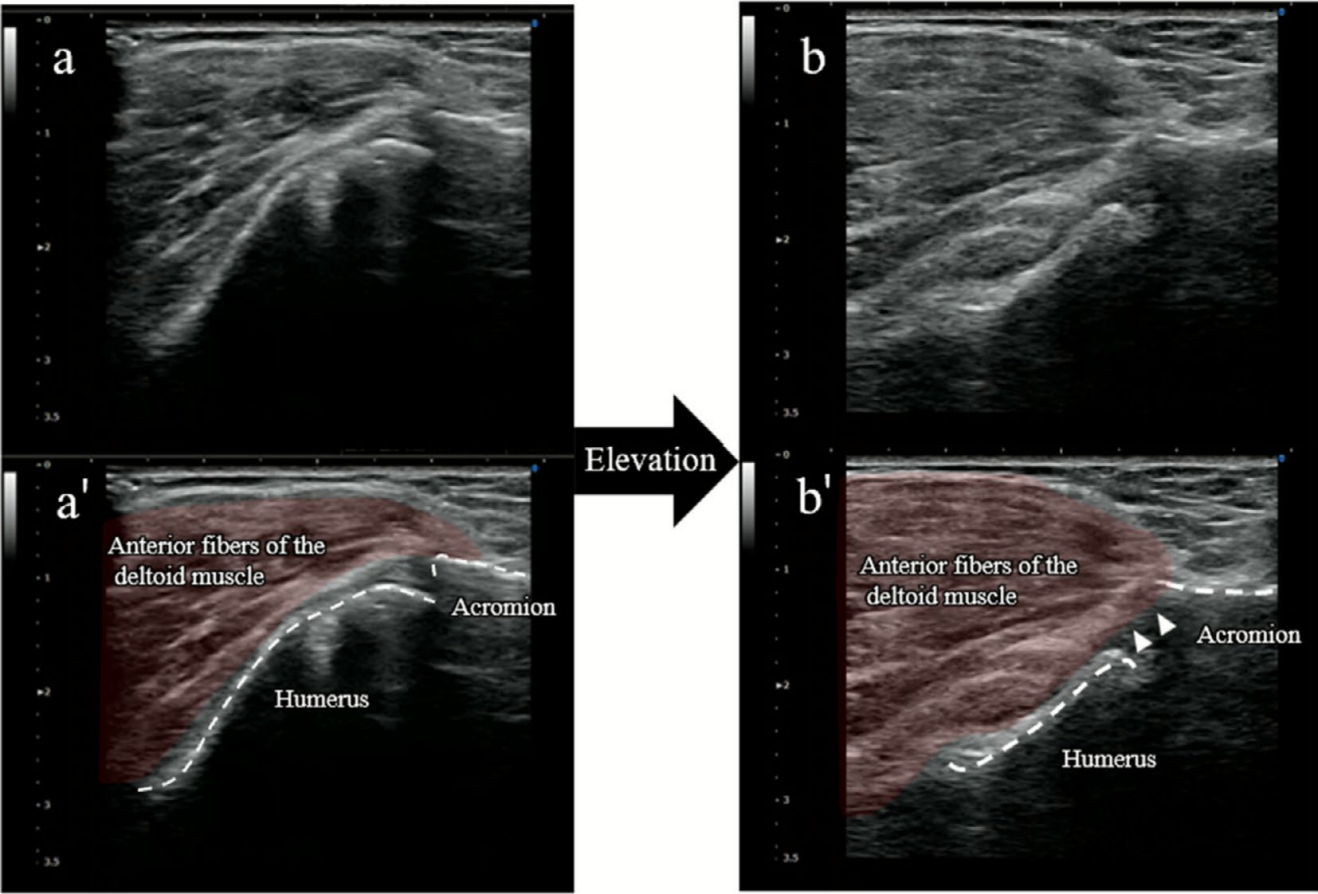
Dynamic ultrasound findings (long‐axis view to the humeral shaft) at the initial evaluation without taping (corresponding to Video S1). Panels a and b show real‐time ultrasound images at rest and forward elevation, respectively. Panels a' and b' show the corresponding schematic illustrations. During forward elevation (b, b'), the humeral head shifts anterosuperior toward the acromion, indicating subacromial impingement (arrowhead).

For dynamic assessment, ultrasound (SONIMAGE HS1; Konica Minolta, Tokyo, Japan) was performed using an 11‐MHz linear probe placed anteriorly over the acromion and humeral head. During forward elevation, the humeral head shifted anterosuperiorly, resulting in subacromial impingement, which was clearly visualized (Figure 2, Video S1).

## 3 Differential Diagnosis, Investigations and Treatment

**FIGURE 3:**
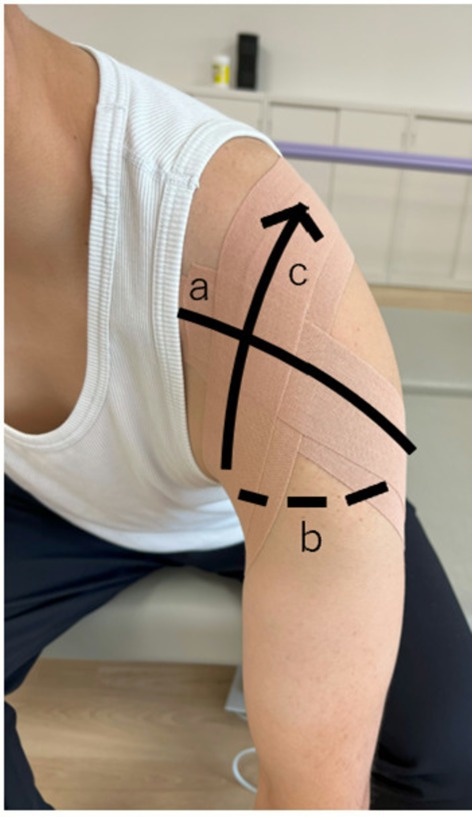
Outline of the figure‐of‐eight stabilizing shoulder taping technique. With the patient in a trunk‐forward position, a single kinesiology tape strip was applied in a continuous figure‐of‐eight pattern. (a) The tape began at the coracoid process and crossed the anterior humeral head, wrapping laterally to posteriorly around the upper arm (approximately 10%–20% elongation). (b) It then progressed horizontally across the posterior upper arm. (c) Finally, it was directed upward from the axilla toward the anterior acromion, again with approximately 10%–20% elongation.

Based on the method reported by Kawabata et al. [7], the patient was positioned with the trunk leaning forward and the arm hanging naturally from the side. Two kinesiology tapes (37.5 mm wide, ~60 cm long each; C&G, Tokyo, Japan) were applied according to the following procedure (Figure 3): Tape tension was estimated clinically as moderate‐to‐high stretch, corresponding to approximately 10%–20% elongation beyond resting length, defined operationally as stretching a 5‐cm tape segment to approximately 5.5–6.0 cm. No skin irritation or intolerance was observed throughout the intervention period.

1. The tape was applied from the coracoid process across the anterior humeral head, extending laterally to posteriorly around the upper arm (approximately 10%–20% elongation).
2. The tape was passed horizontally across the posterior aspect of the upper arm.
3. From the axilla, tape was applied upward across the anterior humeral head toward the anterior acromion, again with approximately 10%–20% elongation.
4. The tape was anchored to the scapular spine posteriorly.

After the trunk was returned to the upright position, the tension of the tape was directed anterosuperiorly to the humeral head, providing enhanced anterior stabilization.

**FIGURE 4:**
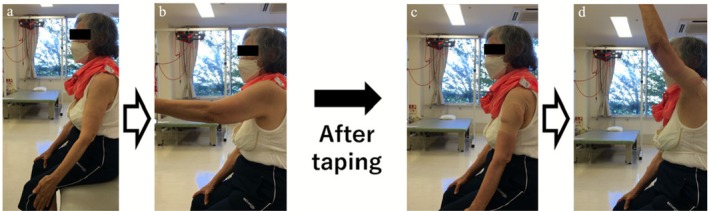
Immediate improvement in forward elevation after taping. (a, b) Before taping, active forward elevation was painful and limited to 85°, with pain rated at numerical rating scale (NRS) 8. (c, d) Immediately after taping, pain decreased to NRS 2, allowing smoother elevation, which improved to 145°.

**FIGURE 5:**
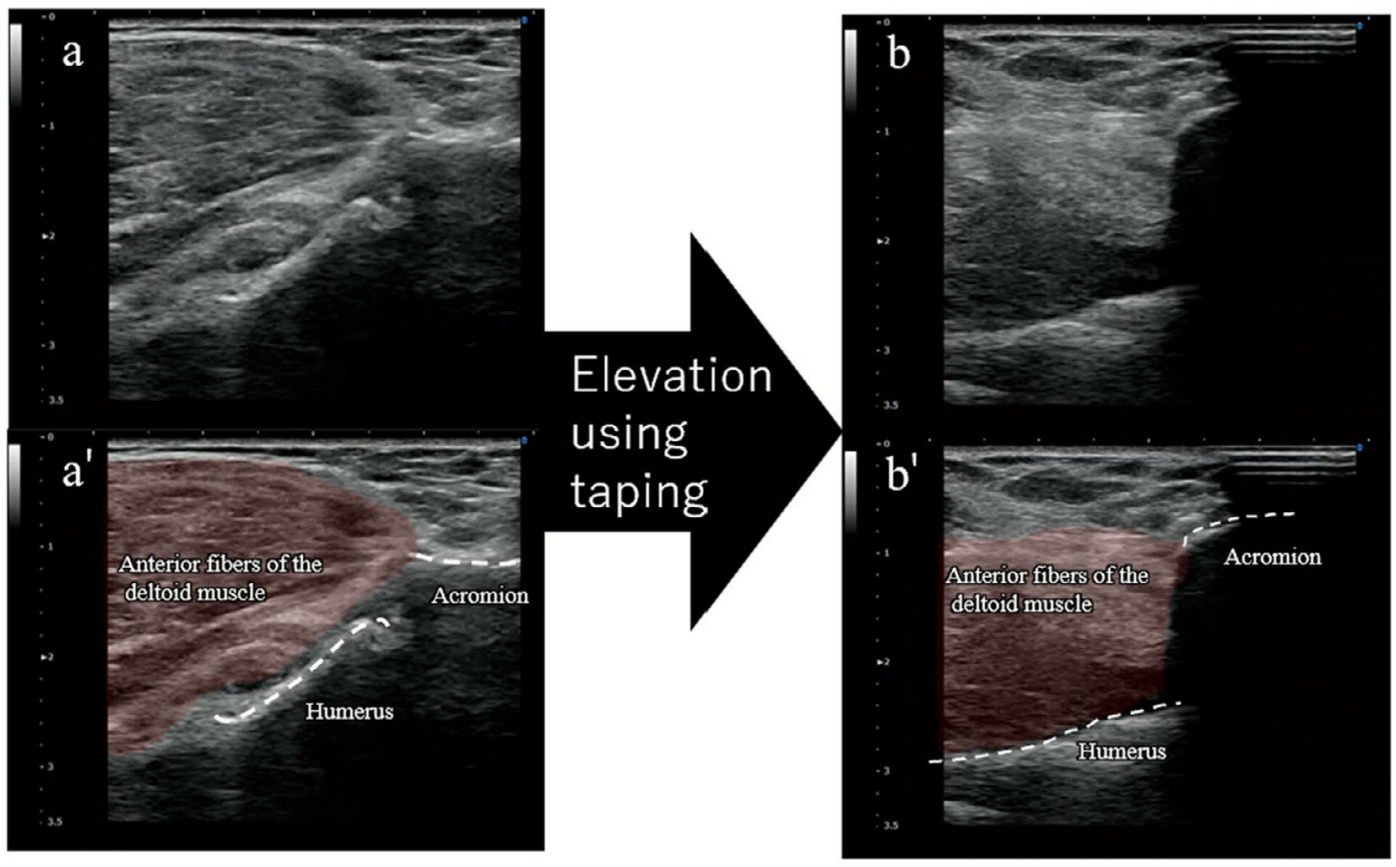
Dynamic ultrasound findings (long‐axis view to the humeral shaft) demonstrating the immediate post‐taping changes observed at the initial evaluation (corresponding to Video S2). Panels a and b show real‐time ultrasound images in the rest and during forward elevation, respectively. Panels a' and b' show the corresponding schematic illustrations. During forward elevation (b, b'), the humeral head translates smoothly beneath the acromion without impingement, enabling greater elevation.

Immediately following application, active forward elevation increased from 85° to 145°, and pain decreased from NRS 8 to 2 (Figure 4). Dynamic ultrasound demonstrated that during forward elevation, the humeral head no longer translated anteriorly or impinged beneath the acromion but instead rotated smoothly and centered beneath the acromion (Figure 5, Video S2).

To sustain and enhance the treatment effect, exercise therapy with real‐time ultrasound feedback was performed while the anterior stability of the shoulder was supported by a figure‐of‐eight stabilizing tape. The taping technique was applied at every rehabilitation session from the initial visit through week 4. Thereafter, the taping frequency was gradually reduced, and no taping was applied after week 8. Because the tape was removed at the end of each rehabilitation session for outcome assessment, the patient performed all home exercises without taping.

**FIGURE 6:**
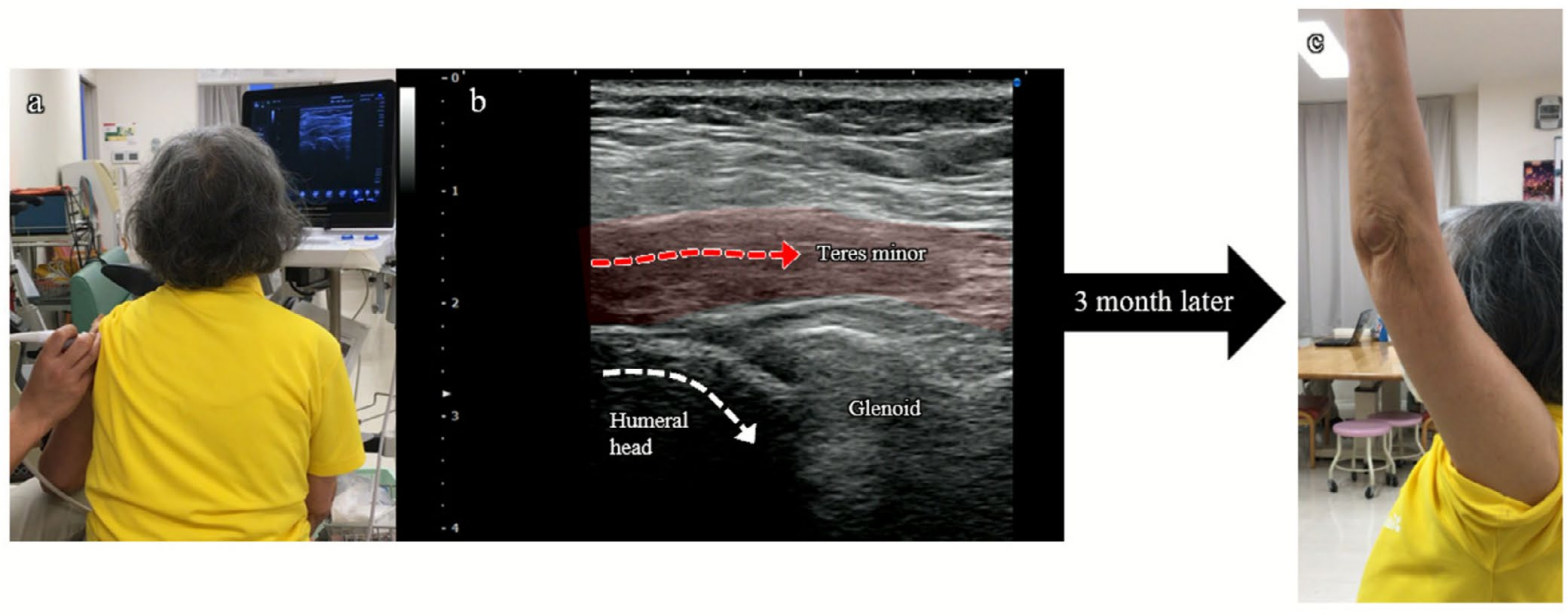
Ultrasound‐guided exercise therapy (long‐axis view to the teres minor muscle) performed under figure‐eight stabilizing shoulder taping. (a) Exercise therapy conducted with real‐time ultrasound feedback while the shoulder was stabilized using figure‐eight stabilizing shoulder taping. (b) Dynamic ultrasound image demonstrating teres minor contraction (red arrow), promoting smooth axial rotation of the humeral head (white arrow) (see also Videos S3 and S4). (c) Clinical outcome at 3 months: active forward elevation improved from 85° to 165°, and pain during motion decreased from NRS 8 to 3.5 without taping.

The aim was to facilitate contraction of the residual cuff muscles, particularly the teres minor and parts of the infraspinatus, while suppressing the excessive compensatory activity of the deltoid. During the intervention, an 11‐MHz linear probe was placed posteriorly over the shoulder to visualize the muscle belly and tendon insertion of the posterior cuff (Figure 6a). The patient was shown alternating images of the unaffected and affected sides, providing visual feedback on posterior cuff contraction. The exercise tasks included external and internal rotations in the resting position and external rotation at approximately 90° of shoulder flexion. For each task, the patient was instructed to achieve selective posterior cuff contraction while minimizing deltoid overactivity (Figure 6b). Each exercise was performed for three sets of 20–30 repetitions. A contraction was considered adequate when visible thickening of the posterior cuff muscle belly and smooth axial rotation of the humeral head were observed on ultrasound, without excessive deltoid activation.

After approximately 5 min of training, the posterior cuff achieved full contraction with smooth humeral head axial rotation. Dynamic changes before and after exercise are shown in Videos S3 and S4. Video S3 shows insufficient contraction with reduced humeral head axial rotation, whereas Video S4 shows increased posterior cuff thickness and improved humeral head rotation following effective contraction. This exercise was performed under the therapist's supervision for 3 months, with 1–2 sessions per week (a total of 23 sessions). The patient was instructed to perform similar home exercises daily, recalling the sensation of cuff contraction.

## 4 Conclusion and Results (Outcome and Follow‐Up)

Baseline and follow‐up assessments without taping included the JOA score (baseline and 3 months) and range of motion and NRS (baseline and 1, 2, and 3 months).

Immediate post‐intervention pain and active elevation were assessed during taping application.

With continued intervention, shoulder range of motion and pain progressively improved every month, and these improvements were maintained even without taping (Figure 6c). At the final 3‐month follow‐up, the JOA shoulder score improved from 38 to 71.5 points (Table 1). Active forward elevation increased from 85° to 165°, and pain on motion decreased from 8 to 3.5 on the NRS without taping. Importantly, the patient was able to return to her desired recreational activity level after 3 months of rehabilitation (Table 2).

**TABLE 1:** Changes in JOA score a 3‐month later intervention period without taping.

Outcome measure	Baseline	3 months
JOA score (/100)	38	71.5

*Note:* JOA: Japanese Orthopedic Association shoulder score (maximum 100 points; assesses pain, function, range of motion, and radiographic findings).

**TABLE 2:** Changes in shoulder function over a 3‐month intervention period without taping.

Outcome measure	Baseline	1 month	2 months	3 months
Forward elevation (°)	85	120	150	165
Abduction (°)	70	90	120	135
Pain on motion (NRS)	8	6	5	3.5

*Note:* NRS: Numerical rating scale for pain (0 = no pain, 10 = worst possible pain).

In this patient with traumatic recurrent MRCT, immediate improvements in pain and active elevation were observed during taping application. With continued rehabilitation, shoulder range of motion, pain, and functional outcomes progressively improved over the 3‐month follow‐up period, and these improvements were maintained even without taping.

## 5 Discussion

In this case, figure‐of‐eight stabilizing shoulder taping was associated with reduced anterior humeral head translation, accompanied by immediate pain relief and improved forward elevation. Dynamic ultrasonography showed smoother humeral head motion during subacromial elevation, which may suggest a possible biomechanical stabilizing effect associated with this taping technique.

Conservative treatment for MRCT may require up to 9 months for anterior elevation to recover from 40° to 160° [9]. In contrast, our patient demonstrated an immediate improvement from 85° to 145°, along with substantial pain reduction, temporally associated with the initial taping intervention. A similar immediate benefit—reduced pain and improved throwing performance—was reported by Kawabata et al. in pitchers with marked anterior instability [7]. Taken together, these observations suggest that this taping method may represent a potential conservative option, even for older adults with traumatic recurrent MRCT, although causal inference cannot be established from a single case.

Dynamic ultrasound further revealed a clear change in humeral head behavior. Before taping, the humeral head impinged beneath the acromion during elevation. After taping, it remained centered throughout the movement. This finding suggests that an anteriorly directed stabilizing force from the tape may be associated with improved joint centering. While previous studies have attributed the effects of kinesiology taping mainly to cutaneous stimulation and proprioceptive feedback [10, 11], our imaging findings may indicate the possibility of an additional biomechanical stabilizing influence on glenohumeral kinematics.

Ultrasound‐guided exercise therapy also played an important role. With the shoulder stabilized by taping, real‐time ultrasound feedback may have facilitated selective activation of the posterior cuff (infraspinatus–teres minor complex) while minimizing excessive deltoid compensation. This muscle group contributes to depressing the humeral head and counteracting deltoid‐induced superior translation [12], yet selective strengthening is notoriously difficult [13]. Similar to its use in trunk muscle re‐education [14], real‐time imaging in this case may have contributed to improved motor awareness and learning, which could be associated with the sustained improvements observed after tape removal.

Clinically, figure‐of‐eight stabilizing shoulder taping was noninvasive, easy to apply, and potentially valuable for patients who are not candidates for RSA. Although RSA remains the gold standard for MRCT [4], comorbidities, surgical risks, or patient preference may limit its use [5, 6]. The functional gains achieved with taping are smaller than those obtained with RSA; however, its low invasiveness and potential diagnostic value are meaningful. Immediate improvements in pain and range of motion after taping may help inform clinical decision‐making regarding the feasibility of conservative treatment.

This case report has several limitations. It describes a single patient, lacks a control group, has a short follow‐up period, and used a taping method that was not standardized in placement, tension, or duration. In addition, the observed clinical improvements occurred in the context of combined interventions, including stabilizing taping, ultrasound‐guided exercise therapy, and a home exercise program, making it difficult to isolate the effect of any single component. However, the immediate improvements in range of motion and pain were temporally associated with the introduction of stabilizing taping and ultrasound‐guided exercise therapy, suggesting that these interventions may have played a clinically meaningful role. These immediate changes may also reflect non‐specific effects, including reduced pain‐related fear, decreased protective muscle guarding, transient modulation of deltoid muscle activity, or placebo‐related responses. Nevertheless, the relative contribution of each component cannot be determined, and additional cases and comparative studies, including those involving RSA, are needed to clarify indications, long‐term outcomes, and optimal taping protocols.

## Author Contributions

**Takaki Aruga:** conceptualization, data curation, formal analysis, investigation, methodology, resources, visualization, writing – original draft. **Masashi Kawabata:** conceptualization, funding acquisition, methodology, project administration, supervision, visualization, writing – review and editing. **Hidenori Yao:** resources, supervision, writing – review and editing.

## Funding

This study was supported by the Japan Society for the Promotion of Science Grant‐in‐Aid (JSPS KAKENHI, grant numbers: JP22K11294).

## Ethics Statement

This study was conducted in accordance with the Declaration of Helsinki and was approved by the Ethics Committee of Hokuso Shiroi Hospital (study number 25‐3).

## Consent

Written informed consent was obtained from the patient for inclusion in this study and for publication of the findings.

## Conflicts of Interest

The authors declare no conflicts of interest.

## Supporting information


**Videos S1–S4:** ccr371932‐sup‐0001‐VideosS1‐S4.zip.

## Data Availability

The authors have nothing to report.

## References

[1] H. Minagawa , N. Yamamoto , H. Abe , et al., “Prevalence of Symptomatic and Asymptomatic Rotator Cuff Tears in the General Population: From Mass‐Screening in One Village,” Journal of Orthopaedics 10 (2013): 8–12, 10.1016/j.jor.2013.01.008. 24403741 PMC3768248

[2] R. H. Hawkins and R. Dunlop , “Nonoperative Treatment of Rotator Cuff Tears,” Clinical Orthopaedics and Related Research 321 (1995): 178–188. 7497666

[3] S. Weber and J. Chahal , “Management of Rotator Cuff Injuries,” Journal of the American Academy of Orthopaedic Surgeons 28 (2020): e193–e201, 10.5435/JAAOS-D-19-00463. 31599763

[4] M. Cavalier , S. Jullion , J. Kany , et al., “Management of Massive Rotator Cuff Tears: Prospective Study in 218 Patients,” Orthopaedics & Traumatology, Surgery & Research 104 (2018): S193–S197, 10.1016/j.otsr.2018.09.007. 30253987

[5] S. C. Kim , I. S. Kim , M. C. Jang , and J. C. Yoo , “Complications of Reverse Shoulder Arthroplasty: A Concise Review,” Clinics in Shoulder and Elbow 24 (2021): 42–52, 10.5397/cise.2021.00066. 33652512 PMC7943379

[6] A. D. Lachance , R. Steika , F. Chessa , J. Lutton , and J. Y. Choi , “Ethical Considerations in Shoulder Arthroplasty in Patients Who Are Obese,” JSES Reviews, Reports, and Techniques 5 (2024): 216–221, 10.1016/j.xrrt.2024.08.012. 40321870 PMC12047556

[7] M. Kawabata , T. Miyata , and K. Miyatake , “A Novel Figure‐Eight Taping Technique for Managing Anterior Shoulder Instability in a Recreational Pitcher: A Case Report,” International Journal of Sports Physical Therapy 20 (2025): 741–748, 10.26603/001c.136406. 40322518 PMC12048363

[8] H. Sugaya , K. Maeda , K. Matsuki , and J. Moriishi , “Repair Integrity and Functional Outcome After Arthroscopic Double‐Row Rotator Cuff Repair: A Prospective Outcome Study,” Journal of Bone and Joint Surgery. American Volume 89 (2007): 953–960, 10.2106/JBJS.F.00512. 17473131

[9] O. Levy , H. Mullett , S. Roberts , and S. Copeland , “The Role of Anterior Deltoid Reeducation in Patients With Massive Irreparable Degenerative Rotator Cuff Tears,” Journal of Shoulder and Elbow Surgery 17 (2008): 863–870, 10.1016/j.jse.2008.04.005. 18718765

[10] H. Y. Chang , K. Y. Chou , J. J. Lin , C. F. Lin , and C. H. Wang , “Immediate Effect of Forearm Kinesio Taping on Maximal Grip Strength and Force Sense in Healthy Collegiate Athletes,” Physical Therapy in Sport 11 (2010): 122–127, 10.1016/j.ptsp.2010.06.007. 21055705

[11] B. L. Riemann and S. M. Lephart , “The Sensorimotor System, Part II: The Role of Proprioception in Motor Control and Functional Joint Stability,” Journal of Athletic Training 37 (2002): 80–84. 16558671 PMC164312

[12] K. Hoshikawa , M. Dominguez , R. L. Lawrence , et al., “Muscle Compensation Strategies to Maintain Glenohumeral Joint Stability in Rotator Cuff Tears: A Cadaveric Study,” Journal of Bone and Joint Surgery. American Volume 107 (2025): 26–35, 10.2106/JBJS.24.00411. 39471245

[13] M. Tsuruike , T. S. Ellenbecker , and C. Lauffenburger , “Electromyography Activity of the Teres Minor Muscle With Varying Positions of Horizontal Abduction in the Quadruped Position,” JSES International 5 (2021): 480–485, 10.1016/j.jseint.2020.12.014. 34136858 PMC8178592

[14] R. Sarafadeen , S. O. Ganiyu , and A. A. Ibrahim , “Effects of Spinal Stabilization Exercise With Real‐Time Ultrasound Imaging Biofeedback in Individuals With Chronic Nonspecific Low Back Pain: A Pilot Study,” Journal of Exercise Rehabilitation 16 (2020): 293–299, 10.12965/jer.2040380.190. 32724788 PMC7365723

