# Supplementary material for: Figure‐of‐Eight Stabilizing Shoulder Taping Combined With Ultrasound‐Guided Exercise Therapy for Traumatic Recurrent Massive Rotator Cuff Tear: A Case Report
Source: Clin Case Rep. 2026 Jan 24;14(2):e71932. doi: 10.1002/ccr3.71932 (PMC12831206; doi:10.1002/ccr3.71932)
Supplement: Supplementary file 1 — Videos S1–S4: ccr371932‐sup‐0001‐VideosS1‐S4.zip. [file CCR3-14-e71932-s001.zip › the transcription file video 1-4.docx]

**Video 1. Dynamic ultrasound findings at initial evaluation without taping.**

Dynamic ultrasonography during active forward elevation at the initial evaluation without taping.

Readers should note the anterosuperior translation of the humeral head toward the acromion during elevation, accompanied by reduced smooth axial rotation and early subacromial contact.

These kinematic features were observed at baseline and provide a reference for comparison with subsequent conditions.

**Video 2. Dynamic ultrasound findings at initial evaluation after taping.**

Dynamic ultrasonography during active forward elevation immediately after application of figure-of-eight stabilizing shoulder taping.

Readers should focus on the reduction in anterosuperior humeral head translation and the more continuous axial rotation beneath the acromion, compared with the untaped condition (Video 1).

These changes were temporally associated with the application of stabilizing taping and may have contributed to the observed improvement in pain and elevation.

**Video 3. Glenohumeral kinematics before exercise.**

Dynamic ultrasonography obtained before ultrasound-guided exercise therapy.

Readers should observe limited thickening of the posterior rotator cuff muscle belly (infraspinatus–teres minor region) during active movement, accompanied by reduced humeral head axial rotation and restricted glenohumeral external rotation.

These findings were associated with insufficient posterior cuff activation at baseline.

**Video 4. Glenohumeral kinematics after exercise.**

Dynamic ultrasonography obtained after ultrasound-guided exercise therapy.

Readers should note increased posterior rotator cuff muscle thickening during contraction, along with improved humeral head axial rotation and smoother external rotation, compared with the pre-exercise condition (Video 3).

These changes were observed in association with real-time ultrasound feedback–based exercise practice and may have contributed to improved glenohumeral kinematics.
