# Supplementary figures and images for: Figure‐of‐Eight Stabilizing Shoulder Taping Combined With Ultrasound‐Guided Exercise Therapy for Traumatic Recurrent Massive Rotator Cuff Tear: A Case Report
Source: Clin Case Rep. 2026 Jan 24;14(2):e71932. doi: 10.1002/ccr3.71932 (PMC12831206; doi:10.1002/ccr3.71932)

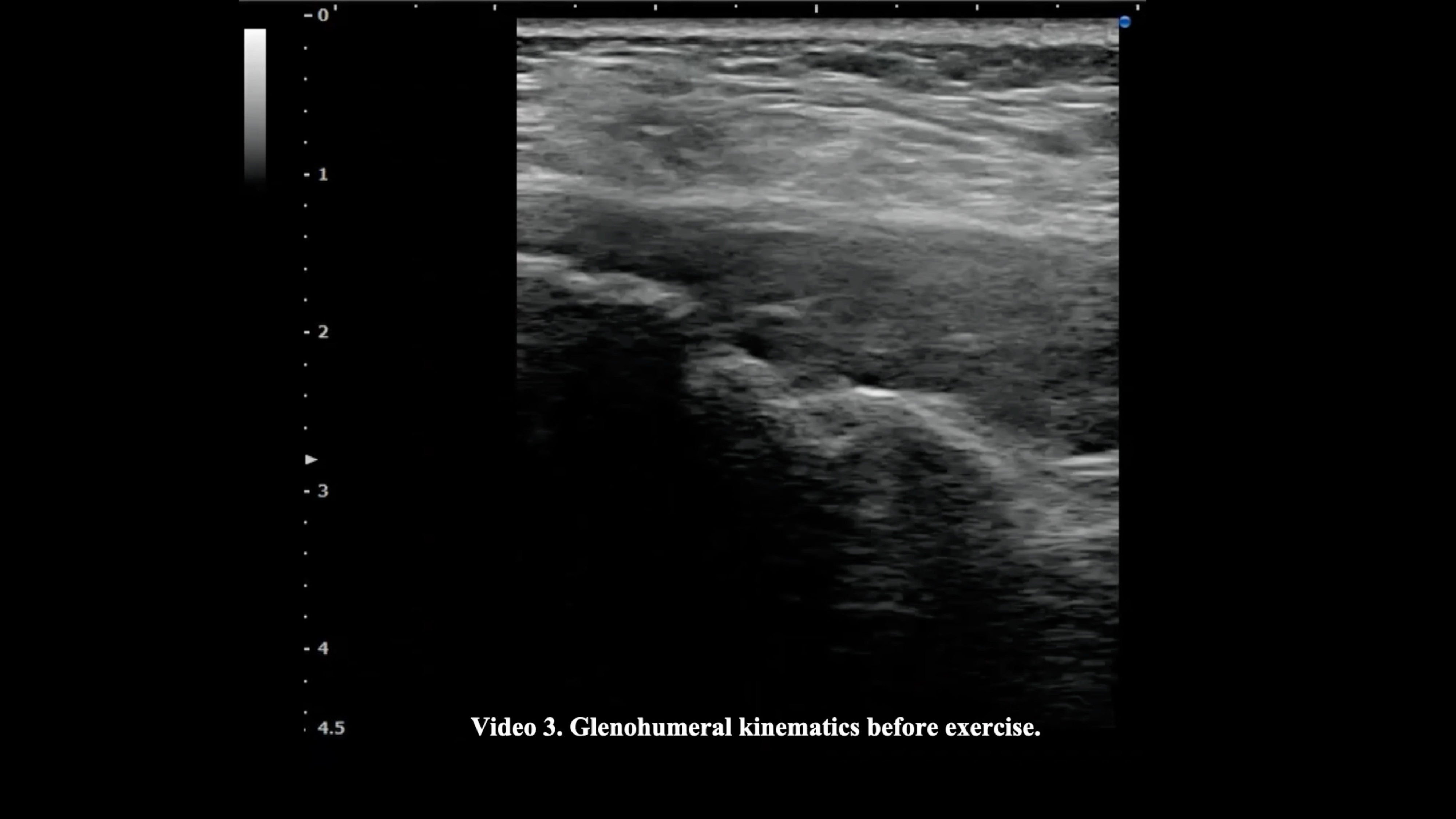

Supplement: Supplementary file 1 — Videos S1–S4: ccr371932‐sup‐0001‐VideosS1‐S4.zip. [file CCR3-14-e71932-s001.zip › Video 3 place holder image.jpg]

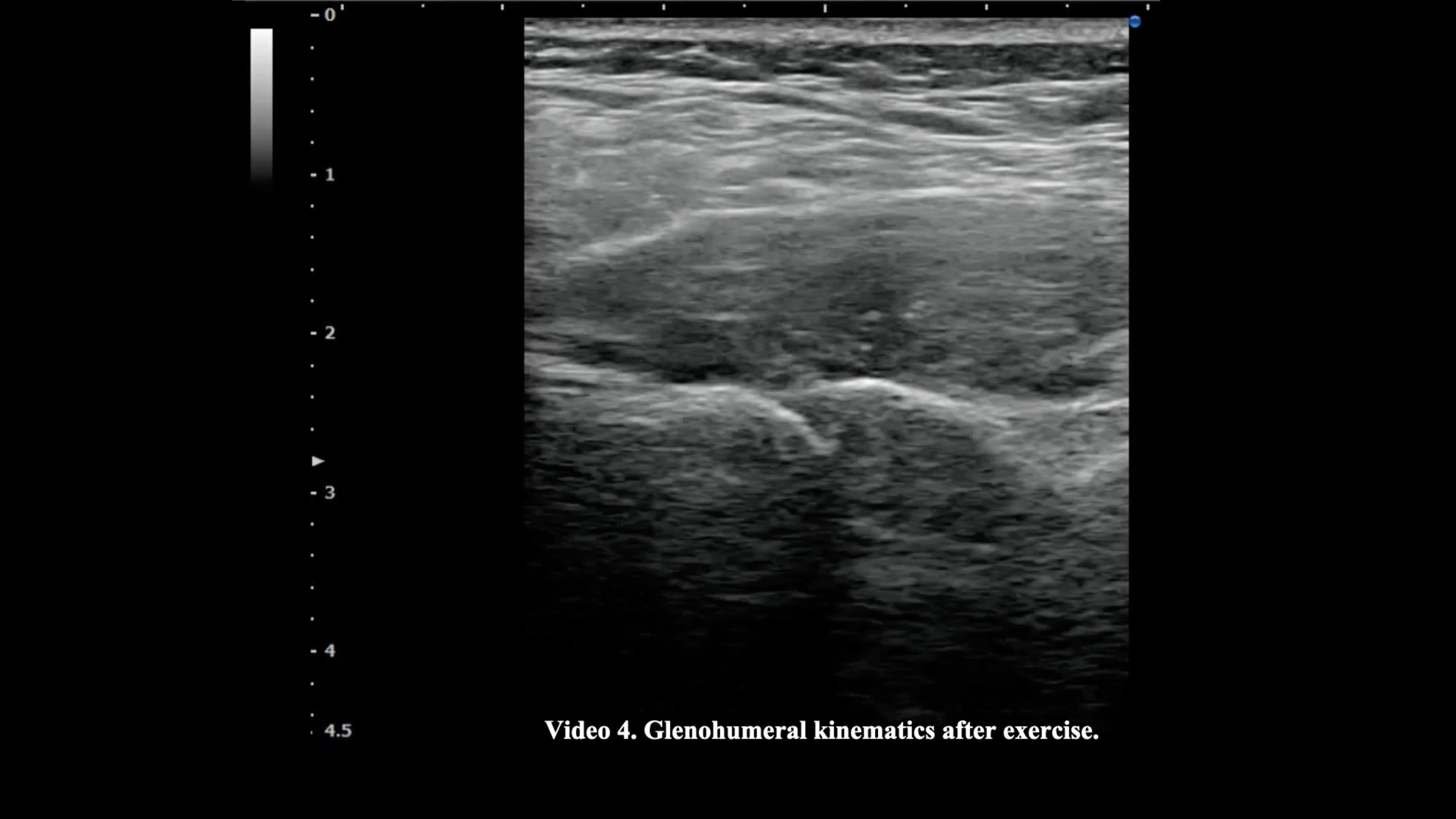

Supplement: Supplementary file 1 — Videos S1–S4: ccr371932‐sup‐0001‐VideosS1‐S4.zip. [file CCR3-14-e71932-s001.zip › Video 4 place holder image.jpg]

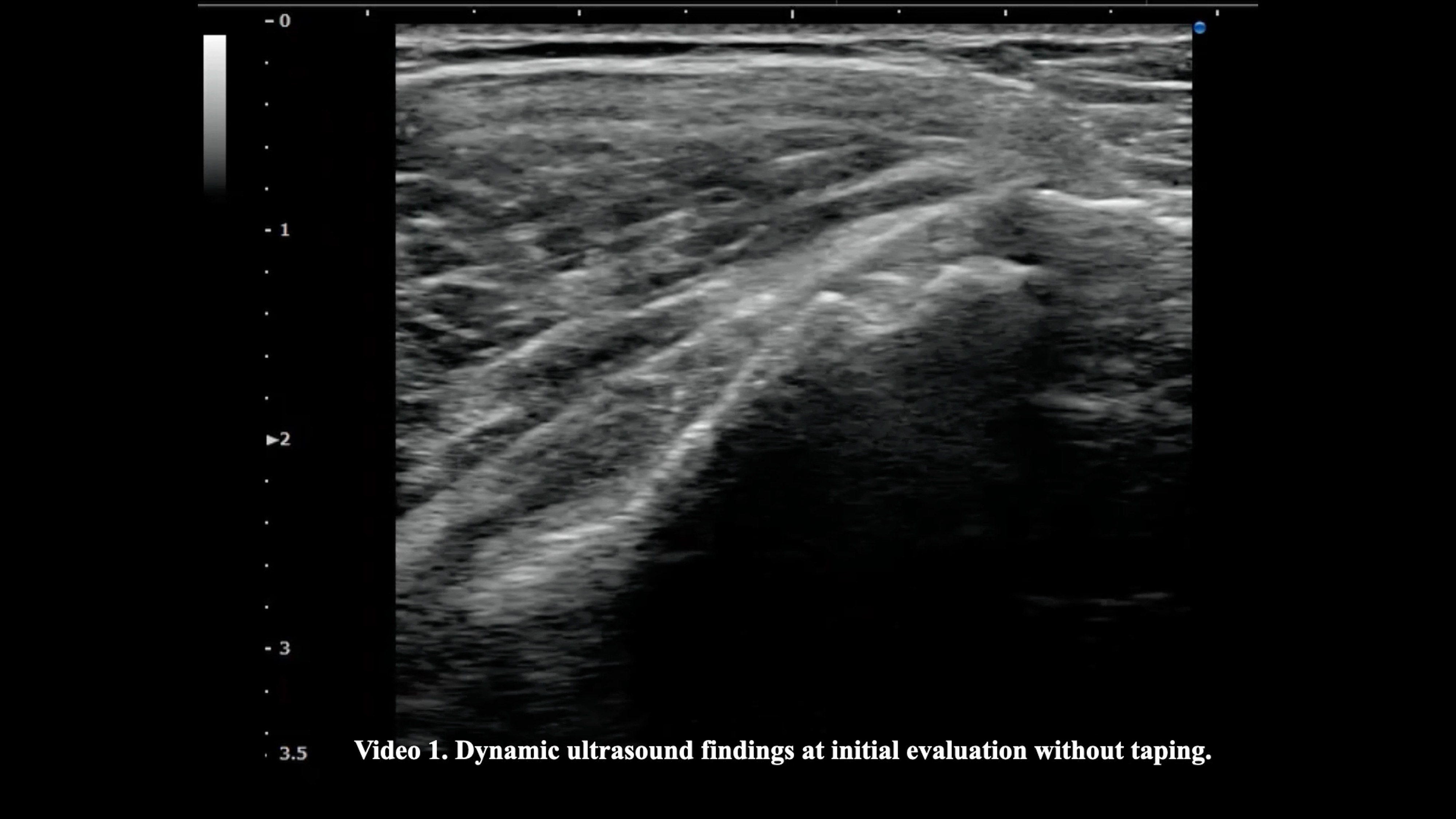

Supplement: Supplementary file 1 — Videos S1–S4: ccr371932‐sup‐0001‐VideosS1‐S4.zip. [file CCR3-14-e71932-s001.zip › Video 1 place holder image.jpg]

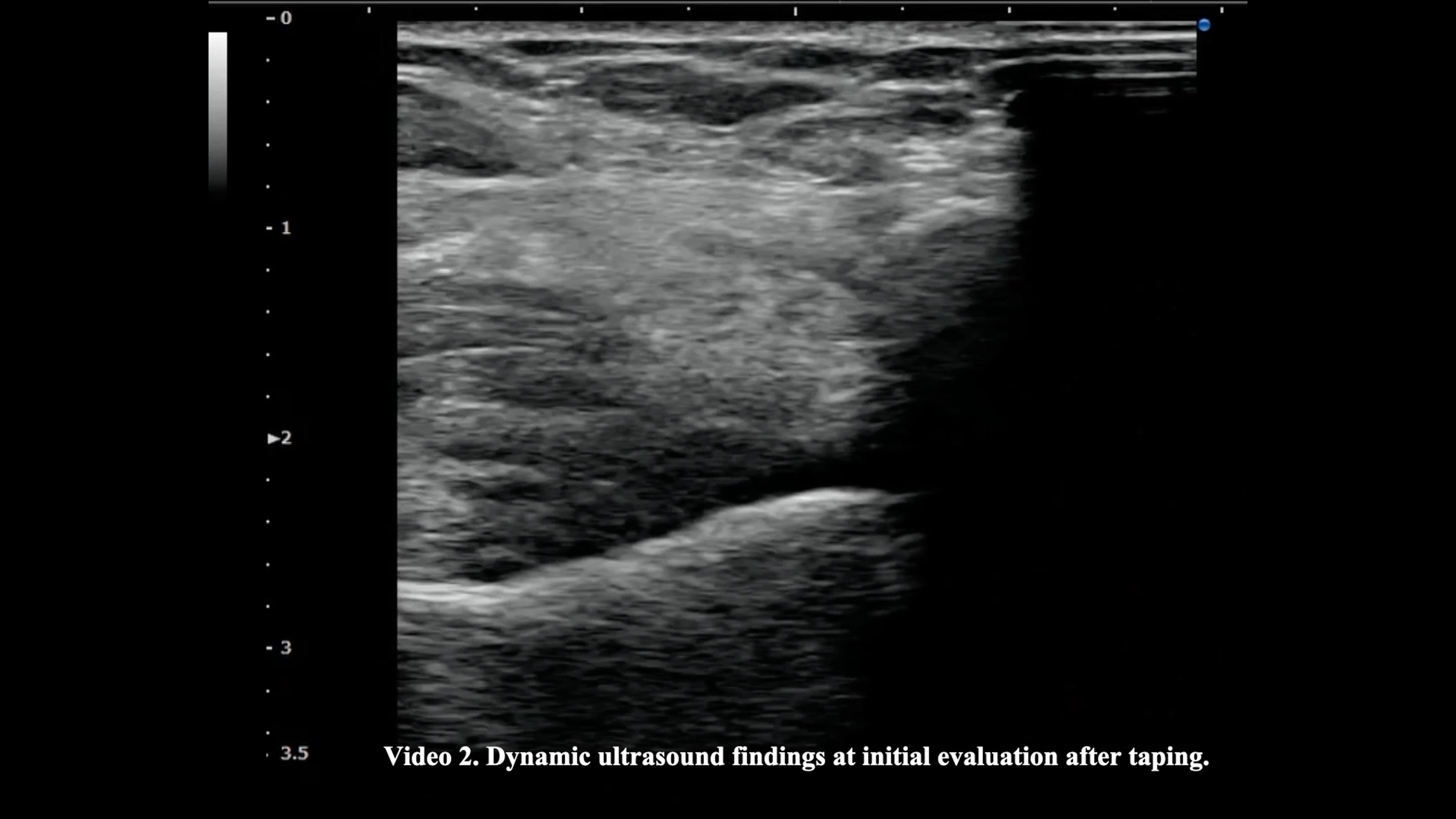

Supplement: Supplementary file 1 — Videos S1–S4: ccr371932‐sup‐0001‐VideosS1‐S4.zip. [file CCR3-14-e71932-s001.zip › Video 2 place holder image.jpg]
